# Occurrence of *Thelazia callipaeda* and its vector* Phortica variegata* in Austria and South Tyrol, Italy, and a global comparison by phylogenetic network analysis

**DOI:** 10.1186/s13071-023-05913-y

**Published:** 2023-08-24

**Authors:** Maria Sophia Unterköfler, Patrick Dengg, Miriam Niederbacher, Sarah Lindorfer, Antonia Eberle, Alexandra Huck, Katalina Staufer, Carina Zittra, Licha Natalia Wortha, Adnan Hodžić, Georg Gerhard Duscher, Josef Harl, Gerhard Schlüsslmayr, Marcos Antonio Bezerra-Santos, Domenico Otranto, Katja Silbermayr, Hans-Peter Fuehrer

**Affiliations:** 1 Institute of Parasitology, University of Veterinary Medicine Vienna, Vienna, Austria; 2 Small Animal Practice Dr. Alexandra Huck, Güttenbach, Austria; 3 Department for Companion Animals and Horses, University of Veterinary Medicine Vienna, Vienna, Austria; 4 Department of Functional and Evolutionary Ecology, University of Vienna, Vienna, Austria; 5 Department of Microbiology and Ecosystem Science, University of Vienna, Vienna, Austria; 6 Institute of Pathology, University of Veterinary Medicine Vienna, Vienna, Austria; 7 Malfattigasse 19, Vienna, Austria; 8 Department of Veterinary Medicine, University of Bari, Valenzano, Italy; 9 Boehringer Ingelheim GmbH, Vienna, Austria

**Keywords:** Oriental eye worm, Canine thelaziosis, Zoophilic fruit fly, Vector-borne disease, Emerging zoonotic disease, *COI*, Cytochrome* c* oxidase subunit I

## Abstract

The zoonotic nematode *Thelazia callipaeda* infects the eyes of domestic and wild animals and uses canids as primary hosts. It was originally described in Asia, but in the last 20 years it has been reported in many European countries, where it is mainly transmitted by the drosophilid fruit fly *Phortica variegata*. We report the autochthonous occurrence of *T.* *callipaeda* and its vector *P.* *variegata* in Austria. Nematodes were collected from clinical cases and fruit flies were caught using traps, netting, and from the conjunctival sac of one dog. Fruit flies and nematodes were morphologically identified and a section of the mitochondrial cytochrome* c* oxidase subunit I gene (*COI*) was analysed. A DNA haplotype network was calculated to visualize the relation of the obtained *COI* sequences to published sequences. Additionally, *Phortica* spp. were screened for the presence of DNA of *T. callipaeda* by polymerase chain reaction. *Thelazia callipaeda* and *P. variegata* were identified in Burgenland, Lower Austria, and Styria. *Thelazia callipaeda* was also documented in Vienna and *P. variegata* in Upper Austria and South Tyrol, Italy. All *T. callipaeda* corresponded to haplotype 1. Twenty-two different haplotypes of *P. variegata* were identified in the fruit flies. One sequence was distinctly different from those of *Phortica variegata* and was more closely related to those of *Phortica chi* and *Phortica okadai*. *Thelazia callipaeda* could not be detected in any of the *Phortica* specimens.

**Graphical Abstract:**

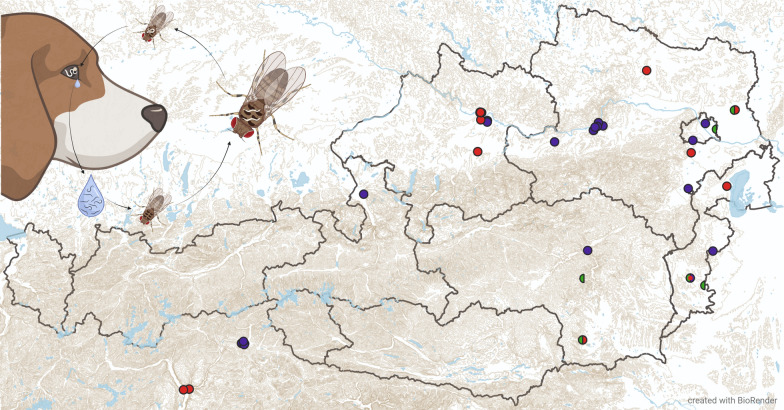

**Supplementary Information:**

The online version contains supplementary material available at 10.1186/s13071-023-05913-y.

## Background

*Thelazia callipaeda* is a parasitic nematode of the order Spirurida that affects the eyes of various mammals and is transmitted by the fruit fly *Phortica variegata*. Among domestic animals, dogs (*Canis lupus familiaris*) are frequently affected, but infections in cats (*Felis silvestris catus*) and rabbits (*Oryctolagus cuniculus*) have also been reported [1–4]. As humans can also be infected, though less frequent, *T. callipaeda* is also a zoonotic nematode and therefore of importance to public health [5, 6]. Among wildlife, *T. callipaeda* has been found in species of the families Canidae, Felidae, Ursidae, Mustelidae, Procyonidae, Suidae, and Leporidae, which represent many possible potential reservoir hosts [7–12].

Clinical signs of infection with *Thelazia callipaeda* can vary widely and have been divided into four stages, ranging from the absence of clinical signs to corneal ulcers [3]. *Thelazia callipaeda* is usually found under the eyelid and the nictitating membrane, and is easily distinguishable from other nematodes that can affect the eye, such as *Onchocerca lupi*, which is embedded in the tissue around the eye and often associated with nodule formation [13]. In combination with the removal of the nematodes from the eye, macrocyclic lactones, such as moxidectin and milbemycin oxime, are useful for both the prevention of infection and its treatment [14–17].

*Thelazia callipaeda* is also known as the ‘oriental eye worm’ due to its original distribution in Asia. In Europe, *T. callipaeda* has been documented in most southern and central countries and is predicted to spread further across the continent [18–20]. The commonly used DNA barcode region, a section of the cytochrome* c* oxidase subunit I gene (*COI*), is useful for the molecular identification of *T. callipaeda* and to distinguish different haplotypes. In the European population of *T. callipaeda*, only one haplotype has been detected, whereas the Asian population is highly diverse, with over 20 different known haplotypes [21, 22].

The sexual reproduction of *T. callipaeda* takes place in mammals, which act as the definitive hosts, while zoophilic fruit flies of the genus *Phortica* are the intermediate hosts. Male *Phortica* spp. feed on lacrimal fluid and take up first-stage larvae of *T. callipaeda* during feeding [22]. In the intermediate host, the larvae can survive for up to 147 days and develop into third-stage larvae, which can be transmitted to a new definitive host when the fruit fly next feeds on lacrimal fluid [23].

In Europe, the main vector of *T. callipaeda* is *P. variegata*, whereas in Asia it is *P.* *okadai* [22]. *Phortica oldenbergi* is also a competent vector under laboratory conditions, but its vector capacity under field conditions needs to be assessed [24]. Dissection and polymerase chain reaction (PCR) can be used to detect larvae of *T. callipaeda* in *Phortica* spp.; however, live fruit flies are necessary for the nematode’s detection through dissection [25, 26].

For the collection of *P. variegata*, fruit fly traps can be hand-made cost efficiently from easily available components. Alternatively, netting placed around the eye of a human or dog can be used for this purpose. Although this is a time-consuming and less efficient method than using fruit fly traps, more male specimens can be collected when using this approach [27]. Identification can be done by using morphological features or by analysing the *COI* barcode region [28–31].

Suitable habitats for *P. variegata*, which are mountainous areas at 600–1200 m above sea level, can be found in large parts of Europe, and in particular central Europe. *Phortica variegata* fruit flies are mainly active at 20–25 °C and their lachryphagous activity increases with air temperature [31, 32].

Few records of *P. variegata* exist for Austria, but recent autochthonous infections of *T. callipaeda* have been reported [33–36]. The few records of *P. variegata* that were available at the start of this study dated from before 1988, and it is not clear if this drosophilid fruit fly is still endemic in Austria and, if it is, how widespread it is [35, 36].

A first report of *P. variegata* in Burgenland, Austria, was published only recently [37], and the genetic diversity of *T. callipaeda* and that of its vector *P. variegata* have not yet been investigated in this region. The aims of this study were to examine whether *P. variegata* and *T. callipaeda* occur in different parts of Austria, and to assess their genetic diversity. The occurrence of *P. variegata* and *T. callipaeda* was also investigated in South Tyrol, which is located in Italy and borders Austria, as this should provide further information on the distribution of these species, which are endemic in some other Italian regions.

## Methods

### Sample collection

A questionnaire designed to identify cases of *T. callipaeda* infections recorded in private veterinary practices was sent to all of the veterinarians in the database of Boehringer Ingelheim (Vienna, Austria) who had given their consent to receive customer mailings. The questionnaire could be completed online between October and November 2020. The veterinarians had the possibility to send the *T. callipaeda* specimens that they had collected previously or from a subsequent clinical case to the Institute of Parasitology, University of Veterinary Medicine Vienna. Material of clinical cases was included for 2015 to 2022, and included that from a published case report [34]. Specimens collected from pets that had previously been abroad were excluded from the study.

**Fig. 1:**
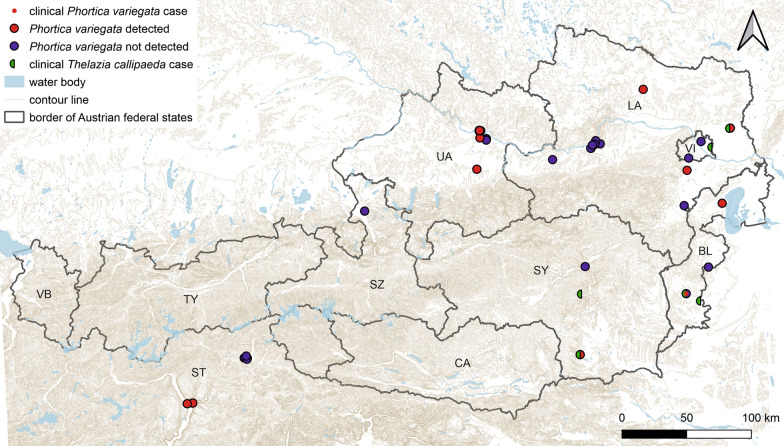
Geographical distribution of sampling sites for *Phortica variegata* and location of the residence of the infected animal, or if not available, that of the clinic of the treating veterinarian of clinical cases of *Thelazia callipaeda* included in this study.* BL* Burgenland,* CA* Carinthia,* LA* Lower Austria,* SZ* Salzburg,* ST* South Tyrol,* SY* Styria,* TY* Tyrol,* UA* Upper Austria,* VI* Vienna,* VB* Vorarlberg. [Map created using QGIS v.3.22.3 (Free Software Foundation, Boston, MA)]

To collect *P. variegata*, fruit fly traps built out of disposable plastic bottles were set up near forests, fruit trees, and dog-walking areas, as described in detail by Roggero et al. [27]. Every 2 weeks, the fruit flies were collected from the nets and frozen at − 20 °C until further analysis, and the chopped fruit which was used as the bait was changed. In July and August 2020, two traps in each of four sites were sampled. Two of the sites were in areas where infection with *T. callipaeda* had been reported (the town of Deutschlandsberg in Styria and the town of Gänserndorf in Lower Austria), and the two other sites were selected independently of known cases (Floridsdorf district, Vienna and Rohr im Kremstal municipality, Upper Austria). In July, August, and September 2021, eighteen traps were set up in Lower Austria, 17 in Upper Austria, 16 in South Tyrol, and 11 at participating veterinary practices that had reported cases of *T. callipaeda* infections, as well as sites provided by other volunteers (Fig. 1).

Additionally, eight fruit flies were collected from the eyes of a dog, and three by netting. Two *P. variegata* found during another study, which took place during the same period of time as the present study [37], were also included.

All of the samples were morphologically identified at the Institute of Parasitology, University of Veterinary Medicine Vienna or at the Department of Veterinary Medicine, University of Bari.

### DNA extraction, PCR amplification, and sequencing

DNA was extracted from whole specimens of *P. variegata* and *T. callipaeda* using the QIAGEN DNeasy Blood and Tissue kit (QIAGEN, Hilden, Germany). Samples were incubated at 56 °C overnight and processed according to the manufacturer’s protocol. To screen *P. variegata* samples for the presence of DNA of *T. callipaeda* and for genetic identification of *T. callipaeda* samples, PCRs targeting 649-base pair (bp) and 674-bp sections of mitochondrial *COI* were performed using the primers COIintF/COIintR [38] and H14FilaCOIFw/H14FilaCOIRv [39], respectively. DNA barcoding of *P. variegata* was done with the primers Lep-F1/LepR1 [40] and LCO1490/HCO2198 [41], which respectively target 665-bp and 658-bp sections of the *COI* gene. One *Phortica* sp. sample, which was genetically different from *P.* *variegata*, was further analysed by targeting a different region of the *COI* gene using the primers UEA7/UEA10 [42]. PCR products were analysed by electrophoresis in 1.8% agarose gels stained with Midori Green Advance DNA stain (Nippon Genetics Europe, Germany). PCR-positive samples were sent to a commercial company (LGC Genomics, Germany) for sequencing using the PCR primers.

### Phylogenetic analysis

For phylogenetic analysis, nucleotide sequences available from GenBank (National Center for Biotechnology Information) and Barcode of Life Data System (BoldSystems) databases were searched with the Basic Local Alignment Search Tool (BLAST) function, using one of the sequences obtained for each organism. In GenBank, the organism group was specified as *Thelazia* (taxid 103826) for the *T. callipaeda* sequences and *Phortica* (taxid 462262) for the *P. variegata* sequences, with the number of maximum target sequences set to 5000. For *P.* *variegata*, only the species belonging to *Phortica* sensu stricto were included. The sequences were aligned and sorted using the default option (FFT-NS-2) in multiple alignment using fast Fourier transform (MAFFT) v.7.311 [43] and sequences not covering the fragment of the sequences obtained in this study were excluded. All sequences featuring obvious sequencing errors and ambiguity characters were removed from the alignment and were excluded from the analysis.

To provide an overview of the diversity of haplotypes, maximum likelihood and Bayesian inference trees were calculated for each organism based on alignments, and included 110 sequences (617 nucleotide positions) for *T. callipaeda* (Additional file 1) and 280 sequences (647 nucleotide positions) for *P. variegata* (Additional file 2). The sequences were collapsed to haplotypes using data analysis in molecular biology and evolution (DAMBE) v.7.0.5.1 [44], leaving 42 haplotypes for *T. callipaeda* and 188 haplotypes for *P. variegata*. A sequence of *Mastophorus muris* (GenBank accession number MK867476) was used as an outgroup for *T. callipaeda*, and a sequence of *Anopheles gambiae* (GenBank accession number MG753768) was used as an outgroup for *P. variegata*. Maximum likelihood bootstrap consensus trees (1000 replicates) were calculated using the W-IQ-TREE web server (http://iqtree.cibiv.univie.ac.at/; [45]) applying the models TIM2 + F + I + G4 for *T. callipaeda* and TIM + F + I + G4 for *P. variegata*, which were suggested as the best fit for the data sets in the model test according to the corrected Akaike information criterion. The Bayesian inference trees were calculated using MrBayes v.3.2.7 [46], applying the next complex model GTR+G+I because the same models were not available in this program. The analyses were run for 10^6^ generations (number of chains, 4), sampling every thousandth tree. The first 25% of the trees were discarded as burn-in and 50% majority-rule consensus trees were calculated based on the remaining 7500 trees.

Based on the results of the consensus tree, clades were selected for the calculation of median-joining haplotype networks using Network 10.2.0.0 (Fluxus Technology, Suffolk, UK), applying the default settings. Networks were graphically prepared and provided with information on the countries and hosts in Network Publisher v.2.1.2.3 (Fluxus Technology) and finalized with CorelDRAW 2021 (Corel, Ottawa, ON).

## Results

In total, the questionnaire was filled out by 183 participating veterinarians. Of these, 16 practitioners stated that they had detected *T. callipaeda* and specified the hosts as dogs (*n* = 11), cats (*n* = 2), and a horse (*n* = 1), from Burgenland (*n* = 5), Lower Austria (*n* = 1), Salzburg (*n* = 1), Styria (*n* = 5), and Vienna (*n* = 1). The report of *Thelazia callipaeda* in a horse came from Carinthia and was assumed to be a misidentification since horses have never been reported as hosts of *T. callipaeda* but rather as hosts of *Thelazia lacrymalis* [47, 48]. In total, 12 *T. callipaeda* specimens from six dogs were collected during the period 2015–2022. The dogs had not travelled abroad prior to diagnosis and originated from Styria (*n* = 2), Lower Austria (*n* = 1), Vienna (*n* = 1), and Burgenland (*n* = 2) (Fig. 1).

*Phortica variegata* (*n* = 45) was detected in five of the seven investigated provinces (Table 1; Fig. 1). Thirty-two specimens were caught in the fruit fly traps; of these, 17 were females, eight were males and seven were unidentified (Table 1). Eight fruit flies were found in the eye of a 2-year-old male Doberman Pinscher from Burgenland (Fig. 1; Table 1). Both eyes of this dog showed ocular discharge, which had started 2 weeks previously. It was treated with a combination compound containing moxidectin (2.5 mg/ kg BW) and imidacloprid (10 mg/kg BW) Spot-On (Advocate; Bayer, Leverkusen, Germany) as well as with a topical ointment containing tobramycin and dexamethasone (Tobradex; Novartis, Basel, Switzerland). The dog continued to show ocular discharge and was presented at the surgery after another week. Further examination revealed the presence of dead fruit flies in the conjunctival sac of both eyes. The flies were removed using cotton swabs, after which the ocular discharge resolved.

**Table 1:** *Phortica variegata* analysed in this study

Province	Female	Male	Sex not determined	Total	Collection site
Burgenland	1	5	2	8	Dog eye
Burgenland	0	1	0	1	[37]
Lower Austria	0	1	0	1	[37]
Lower Austria	0	1	2	3	Netting
Lower Austria	0	0	1	1	Traps
Styria	2	0	0	2	Traps
South Tyrol	10	6	5	21	Traps
Upper Austria	5	2	1	8	Traps
Total	18	16	11	45	

The sequences obtained in this study were uploaded to BoldSystems (process identifiers PAVEA165-22–PAVEA176-22, PAVEA183-22, PAVEA184-23–PAVEA227-23) and GenBank (accession numbers OP620892–OP620903, OQ507612, OQ359791–OQ359834, and OQ689078).

**Fig. 2:**
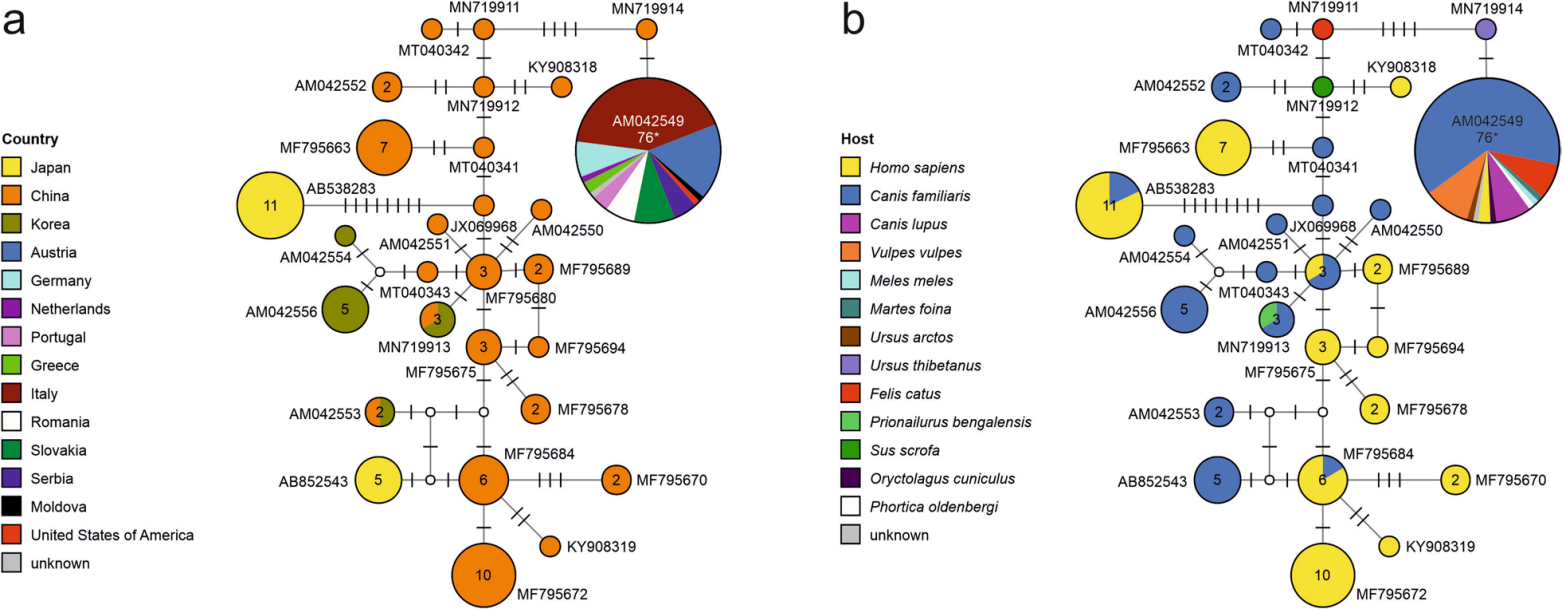
Median-joining haplotype network of the cytochrome* c* oxidase subunit I gene (*COI*) sequences (617 nucleotide positions) of *Thelazia callipaeda* showing the geographical distribution (**a**) and the reported hosts (**b**). Circles represent haplotypes; numbers within the circles represent the number of individuals; if no number is shown, then only one individual is represented. Representative GenBank accession numbers of the haplotypes are shown next to the circles; white circles represent intermediate nodes; bars on branches connecting haplotypes represent the number of substitutions; asterisks indicate haplotypes of the individuals obtained in the present study

**Fig. 3:**
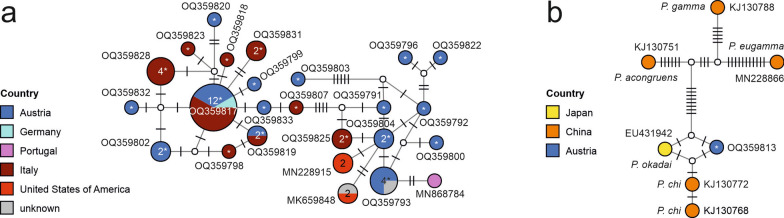
Median-joining haplotype network of the *COI* sequences (647 nucleotide positions) of *Phortica variegata* sensu stricto (**a**) and *Phortica* spp. closely related to the unknown specimen from the present study (**b**) showing the geographical distribution. Circles represent haplotypes; numbers within the circles represent the number of individuals; if no number is shown, then only one individual is represented. Representative GenBank accession numbers of the haplotypes are shown next to the circles; white circles represent intermediate nodes; bars on branches connecting haplotypes represent the number of substitutions; asterisks indicate haplotypes of the individuals obtained in the present study

All *T. callipaeda* corresponded to haplotype 1, which is the only haplotype that has been found in Europe so far (Fig. 2). In the fruit flies, 43 sequences could be assigned to *P. variegata*, with a total of 22 different haplotypes. In the case of one fruit fly from a dog’s eye, it was not possible to obtain a sequence of sufficient quality and it was therefore excluded from the phylogenetic analysis. One sequence was different from those of *P. variegata* and more similar to those of species previously found in Asia, such as *Phortica chi* and *Phortica okadai* (Fig. 3). Analysis of this sample using a different region of the *COI* gene showed 99.67% similarity to one *P. okadai* (GenBank accession number EU431942), 92.16% similarity to another *P. okadai* (GenBank accession number: EF576924), and only 93.46% and 92.32% similarity to *Phortica variegata* and *Phortica semivirgo*, respectively (GenBank accession numbers MK659848 and EF576935, respectively). *Thelazia callipaeda* could not be detected in any of the *Phortica* specimens.

## Discussion

*Thelazia callipaeda* and its vector *P. variegata* were found in different parts of Austria in the present study. Although *P. variegata* had been previously reported from Burgenland, Lower Austria, Styria, and Vienna [35–37], it was detected for the first time here in Upper Austria. New areas in the distributions of *P. variegata* and *T. callipaeda* were identified in the present study, but the presence and absence of the parasite in Austria could not be seamlessly mapped, as not all Austrian provinces were sampled and the locations of the traps were based on the presumed suitability of the habitats for the host species and not according to a systematic grid.

Female *P. variegata* were mainly caught in the fruit fly traps, and males predominantly around the eyes of the dogs. The preference of male *P. variegata* for the eye was not unexpected as this has also been observed in other studies, and only male *P. variegata* are considered to act as vectors of *T. callipaeda* [25, 27, 49]. To the best of our knowledge, clinical signs caused by the presence of *P. variegata* fruit flies in the conjunctival sac of a dog have not been reported up until now. It is likely that the obtained fruit flies were caught in the eyes while feeding on lacrimal fluid.

As expected, only *T. callipaeda* haplotype 1, which is the only haplotype detected in Europe to date, was found in Austria. In contrast, there is a high haplotype diversity in Asia [21, 50]. It is presumed that *T. callipaeda* is not native to Europe and that its introduction into Europe occurred as a single event. This hypothesis is also supported by the fact that *T. callipaeda* was first reported in Italy in 1989, after which it spread to other European countries due to the presence of *P.* *variegata*, which acts as an intermediate host [25, 51–53].

In both Europe and Asia, *T. callipaeda* is commonly found in dogs, although several wild animals have also been reported as hosts in Europe. Interest in this parasite has increased since its presence in Europe was first reported, and reports of it presence in new hosts are probably partly due to increased research efforts. Recent reports indicate that it is likely that wild animals in Asia are also frequently infected [10, 11]. Although there are case reports of human infections with *T. callipaeda* in Europe, these are more common in Asia. The infection rate in animals in Europe is probably not yet high enough to lead to many human cases. However, this may change in the future if this parasite becomes more prevalent in Europe [3, 18]. That the current prevalence of *T. callipaeda* in Austria is probably low was indicated by the low number of reported clinical cases of thelaziosis in the present study and the fact that none of the investigated *Phortica* fruit flies were positive for this parasite.

Many species within the *P. variegata* complex are not monophyletic at the *COI* barcoding region. However, *P. variegata *sensu stricto was shown to be monophyletic in both a previous study [54] and in the present one. Two haplotypes of *P. variegata* have been reported in the USA and 23 different haplotypes in Europe, including the 20 new ones reported in this study. The diversity of haplotypes found in Austria can be attributed to the fact that this fruit fly has long been native to Europe [35].

The *COI* barcoding sequence that differed from the sequence of *P. variegata* was more closely related to those of *P. chi* and *P. okadai*, which have not yet been reported from Europe. These latter two species are not monophyletic or clearly separated from their closely related morphospecies or cryptic species, and therefore delineating them through use of the *COI* gene is limited [54, 55]. *Phortica chi* and *P. okadai* have only been reported from Asia, but *P. semivirgo*, another species of the *P. variegata* complex, has been found in Europe [29, 56, 57]. Since no reference sequence of the *COI* barcode region was available at the time of analysis, another region of the *COI* gene was additionally analysed to determine whether the sample might be from a *P. semivirgo* specimen. While the sequence was 99.67% similar to one reported *P. okadai* sequence (GenBank accession number EU431942) it was not closely related to one reported for *P. semivirgo* (GenBank accession number EF576935), and was only 92.16% similar to the *P. okadai* sequence (GenBank accession number EF576924) used in a phylogenetic study comparing European *Phortica* spp. [29].

## Conclusions

Further analysis of *Phortica* spp. with the use of additional genetic markers is needed to clarify the significance of the new sequence found in the present study and to assess its occurrence in other parts of Europe. *Thelazia callipaeda*, as well as its vector *P.* *variegata*, can be considered endemic in Austria.

### Supplementary Information

**Additional file 1: **Bayesian interference (BI) tree featuring mitochondrial cytochrome* c* oxidase subunit I gene (*COI*; 617 nucleotide positions) sequences of *Thelazia *spp. Nodes are marked with BI posterior probabilities and maximum likelihood bootstrap values. Clades which are marked in red were used for calculation of the median-joining haplotype (hpt) network containing the sequences obtained in this study. Scale bar indicates the expected mean number of substitutions per site according to the model of sequence evolution applied.

**Additional file 2: **BI tree featuring *COI* (647 nucleotide positions) sequences of *Phortica *sensu stricto. Nodes are marked with BI posterior probabilities and maximum likelihood bootstrap values. Clades which are marked in red were used for calculation of the median-joining hpt network containing the sequences obtained in this study. Scale bar indicates the expected mean number of substitutions per site according to the model of sequence evolution applied.

## Data Availability

Not applicable.
