# Supplementary material for: Occurrence of Thelazia callipaeda and its vector Phortica variegata in Austria and South Tyrol, Italy, and a global comparison by phylogenetic network analysis
Source: Parasit Vectors. 2023 Aug 24;16:294. doi: 10.1186/s13071-023-05913-y (PMC10464191; doi:10.1186/s13071-023-05913-y)
Supplement: Supplementary file 1 — Additional file 1: Bayesian interference (BI) tree featuring mitochondrial cytochrome c oxidase subunit I gene (COI; 617 nucleotide positions) sequences of Thelazia spp. Nodes are marked with BI posterior probabilities and maximum likelihood bootstrap values. Clades which are marked in red were used for calculation of the median-joining haplotype (hpt) network containing the sequences obtained in this study. Scale bar indicates the expected mean number of substitutions per site according to the model of sequence evolution applied. [file 13071_2023_5913_MOESM1_ESM.pdf]

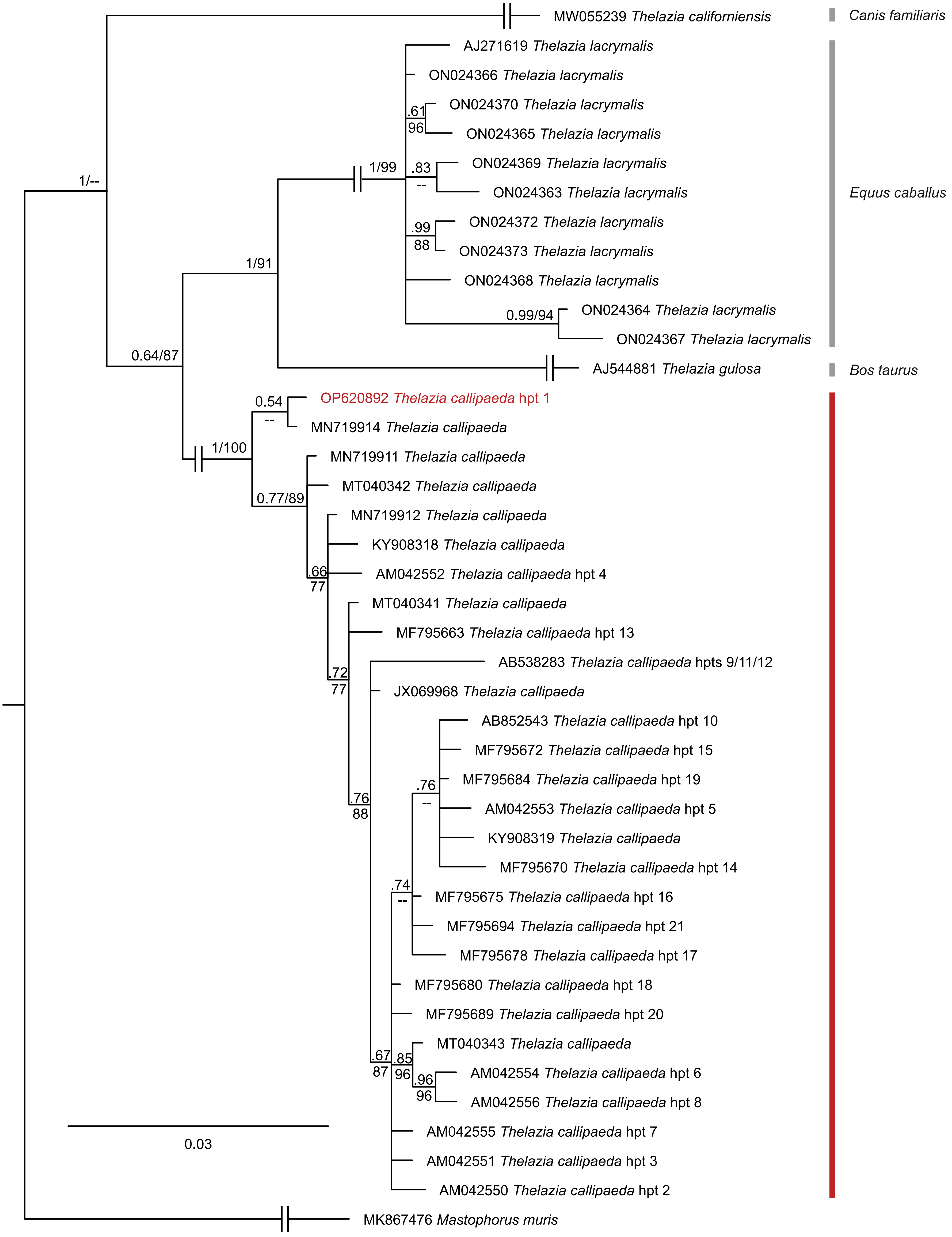

Supplement 1. Bayesian interference tree featuring mitochondrial *cytochrome c oxidase subunit I* gene (617 nucleotide positions) sequences of *Thelazia* spp. Nodes are marked with Bayesian interference posterior probabilities and Maximum likelihood bootstrap values. Clades which are marked in red were used for calculation of the median-joining haplotype (hpt) network containing the sequences obtained in this study. Scale bar indicates the expected mean number of substitutions per site according to the model of sequence evolution applied.
