# Supplementary material for: Occurrence of Thelazia callipaeda and its vector Phortica variegata in Austria and South Tyrol, Italy, and a global comparison by phylogenetic network analysis
Source: Parasit Vectors. 2023 Aug 24;16:294. doi: 10.1186/s13071-023-05913-y (PMC10464191; doi:10.1186/s13071-023-05913-y)
Supplement: Supplementary file 2 — Additional file 2: BI tree featuring COI (647 nucleotide positions) sequences of Phortica sensu stricto. Nodes are marked with BI posterior probabilities and maximum likelihood bootstrap values. Clades which are marked in red were used for calculation of the median-joining hpt network containing the sequences obtained in this study. Scale bar indicates the expected mean number of substitutions per site according to the model of sequence evolution applied. [file 13071_2023_5913_MOESM2_ESM.pdf]

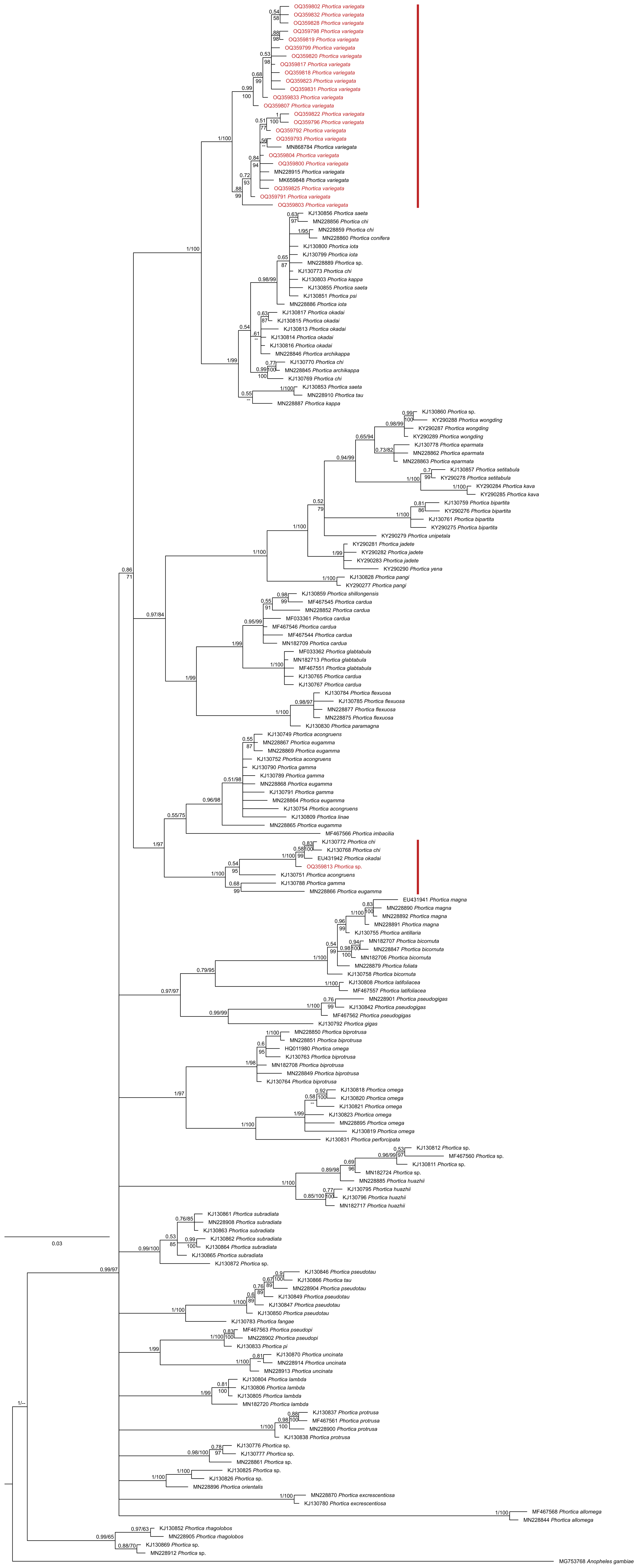

Supplement 2. Bayesian interference tree featuring mitochondrial *cytochrome c oxidase subunit I* gene (647 nucleotide positions) sequences of *Phortica* sensu stricto. Nodes are marked with Bayesian interference posterior probabilities and Maximum likelihood bootstrap values. Clades which are marked in red were used for calculation of the median-joining haplotype (hpt) network containing the sequences obtained in this study. Scale bar indicates the expected mean number of substitutions per site according to the model of sequence evolution applied.
